# Thiol-Functionalized MIL-100(Fe)/Device for the Removal of Heavy Metals in Water

**DOI:** 10.1021/acs.inorgchem.3c01544

**Published:** 2023-11-18

**Authors:** D.R. Sáenz-García, Andreu Figuerola, Gemma Turnes Palomino, Luz O. Leal, Carlos Palomino Cabello

**Affiliations:** † Environment and Energy Department, Advanced Materials Research Center, (CIMAV) S.C., Miguel de Cervantes 120, Chihuahua, Chih. 31136, Mexico; ‡ Department of Chemistry, University of the Balearic Islands, Cra. Valldemossa Km 7.5, Palma 07122, Spain

## Abstract

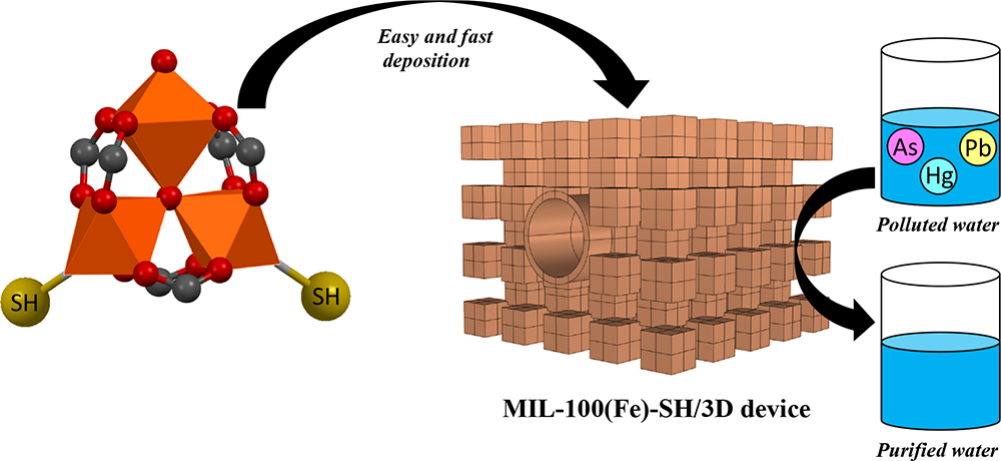

The preparation of a functional device based on a functionalized MIL-100(Fe) metal–organic framework for the solid-phase extraction of heavy metals is reported. By a simple and easy straightforward grafting procedure, a thiol-functionalized MIL-100(Fe) material (MIL-100(Fe)-SH) with a S/Fe ratio of 0.80 and a surface area of 840 m^2^ g^–1^ was obtained. MIL-100(Fe)-SH exhibited a higher Hg(II) extraction (96 ± 5%) than that of MIL-100(Fe) (78 ± 4%) due to the interaction between thiol groups and Hg(II) ions. For practical applications, the obtained MIL-100(Fe)-SH was integrated by a simple method to a 3D printed support based on a matrix of interconnected cubes using poly(vinylidene fluoride) as binder, obtaining a functional device that simultaneously acts as stirrer and sorbent. The developed device showed high efficiency for the removal of Hg(II), good reusability, and excellent performance for the simultaneous preconcentration and further detection and quantification of Hg(II), Pb(II), and As(V) in tap, well, and lake water samples.

## Abstract 2

The functional MIL-100(Fe)-SH/device, which simultaneously acts as stirrer and sorbent, showed excellent performance for the solid-phase extraction of Hg(II), Pb(II), and As(V) in real water samples.

## 1 Introduction

Access to safe and clean potable water is essential for humans and wildlife. Water pollution with a variety of different pollutants, including pathogens, persistent organic pollutants, and heavy metals, has become a global environmental problem.^1^ Among them, heavy metals, which are metals and metalloids with a relative density equal or superior to 5 g cm^–3^,^2^ are a cause of growing concern due to their high toxicity and adverse effects on aquatic biota and human health even at low levels.^3^ Because of their continuous release into the environment from different natural and anthropogenic sources and their persistent and nonbiodegradable nature, different heavy metals have been detected in groundwater, seawater and wastewater.^4−6^ Several traditional water treatments, such as chemical precipitation,^7^ chlorination,^8^ ion exchange,^9^ and membrane filtration,^10^ have been used to remove heavy metals from water. However, some of these methods are ineffective or expensive or generate toxic residues. In this context, the use of porous solids as sorbents has emerged as a simple and effective alternative in the removal of heavy metals from water.^11−13^ Owing to their unique characteristics, such as outstanding porosity, extreme versatility and tunability, thermal and chemical stability, and abundant active sites, metal–organic frameworks (MOFs) have shown great potential as advanced sorbents of different pollutants, including heavy metals.^14−16^ Between the different types of MOFs synthesized so far, the Materials of Institute Lavoisier frameworks (MILs), composed of trivalent metal ions and carboxylic acid groups, are among the most studied for these and other applications thanks to their excellent surface area and water stability.^17^

It has been reported that the incorporation of functional groups on MOFs via functionalization of their linkers and/or metal nodes is a promising approach to improve their extraction capacity of heavy metals.^18^ For instance, an amino-functionalized MIL-101(Cr) was prepared through the coordination of the open Cr^3+^ sites with ethylenediamine and used for Pb^2+^removal.^19^ The Pb^2+^ adsorption capacity of the obtained material was 81 mg g^–1^, which was more than 5 times that of MIL-101 without amino groups. Following a similar approach, a sulfonic acid grafted Cu-MOF, Cu_3_(BTC)_2_-SO_3_H, was obtained and used for the elimination of Cd^2+^ ions, obtaining a high cadmium uptake capacity of 88.7 mg g^–1^.^20^ Using 2-aminoterephthalic acid as a ligand, an NH_2_-MIL-53(Al) MOF was also synthesized and tested for the adsorption of Hg^2+^, showing excellent extraction capacity due to the strong interaction between −NH_2_ groups and Hg^2+^ ions.^21^ More recently, polyethyleneimine modified UiO-66-NH_2_ nanoparticles with excellent Pb^2+^ adsorption capacity, which was attributed to the large amount of −NH_2_ and −NH– groups of polyethyleneimine molecules, were reported.^22^ The potential use of thiol-functionalized HKUST-1 and UiO-66 as sorbents for the removal of Hg^2+^ ions have been also demonstrated.^23^

However, the powder nature of MOF materials limits its applicability for the removal of pollutants from water because tedious and lengthy procedures are necessary to separate the sorbent from the treated water. To overcome this problem and improve the performance of MOFs, they have been immobilized on different supports such as membranes,^24^ monoliths,^25^ sponges,^26^ and fibers.^27^ In this context, 3D printing technology has appeared as an interesting and useful alternative for the fabrication of supports with complex structures in short periods of time.^28−30^ Moreover, not long ago, the combination of MOFs with 3D printing technology has allowed the development of different functional devices for water treatment.^31−34^

In this work, we report the preparation of thiol-functionalized MOF-coated 3D printed devices for the removal of heavy metals from wastewaters. The water-stable and ecofriendly MIL-100(Fe) MOF was functionalized by an easy grafting process, obtaining a thiol-functionalized material (MIL-100(Fe)-SH). The MIL-100(Fe)-SH MOF was applied to the removal of mercury from water, and the performance was compared with the nonfunctionalized MIL-100(Fe). To improve its applicability as an adsorbent, the obtained material was incorporated on a 3D printed device by a fast-coating procedure, obtaining a functional device that simultaneously acts as stirrer and sorbent. The influence of pH on the sample solution and contact time on the removal of Hg(II) was studied. In addition, the potential of the developed device for simultaneous removal of Hg(II), Pb(II), and As(V) from water samples was also evaluated.

## 2 Experimental Section

### 2.1 Instrumentation

XRD diffraction and XRD microdiffraction patterns were acquired utilizing Cu Kα radiation on Siemens D5000 and Bruker D8 diffractometers, respectively. A simulated pattern of bulk MIL-100 was obtained from the crystallographic data reported in the literature^35^ using the Mercury V.3.10.3 software. Thermogravimetric analysis (TGA) was carried out in a nitrogen atmosphere using a TA Instruments SDT 2960 simultaneous DSC-TGA. N_2_ adsorption–desorption isotherms were acquired at 77 K using a TriStar II (Micromeritics) gas adsorption instrument. The samples were previously outgassed under a dynamic vacuum at 423 K for 12 h. Scanning electron microscopy (Hitachi S-3400N) equipped with a Bruker AXS Xflash 4010 EDS system was used to study the morphology and elemental composition of the samples. Infrared spectroscopy (FTIR) using CO as a probe molecule was performed with a Bruker Vertex 80v spectrophotometer equipped with an MCT detector operating at 3 cm^–1^ resolution. For that, the samples were prepared and activated inside the IR cell under a high vacuum at 423 K for 8 h.

### 2.2 Chemicals

All reagents were of analytical grade. Hydrochloric acid (HCl), potassium hexacyanoferrate(III) (K_3_[Fe(CN)_6_]), and potassium hydroxide (KOH) were purchased from J.T. Baker. Iron powder (Fe), poly(vinylidene fluoride) (PVDF, MW ∼180,000), potassium borohydride (KBH_4_), toluene, *p*-benzoquinone, and ammonium hydroxide (NH_4_OH) were acquired from Sigma Aldrich. Trimesic acid (H_3_BTC) and 1,2-ethanedithiol were obtained from Tokyo Chemical Industries. Stock solutions of 1000 mg L^–1^ of each studied metal, *N*,*N*-dimethylformamide (DMF), nitric acid (HNO_3_), and ethanol were purchased from Scharlab. The aqueous solutions used for the extraction experiments were prepared with ultrapure water through a Milli-Q water purification apparatus.

### 2.3 Synthesis of MIL-100(Fe)

Iron-based MIL-100 was prepared at room temperature following a simple procedure reported in the literature.^36^ Basically, a mixture of 0.28 g of Fe, 0.21 mL of HNO_3_, and 25 mL of deionized water was sonicated for 15 min. Later, 0.7 g of trimesic acid and 5.4 mg of *p*-benzoquinone were introduced, and the mixture was left under continuous stirring for 12 h. After this time, 12.5 mL of DMF was added and stirred for 2 h more. Lastly, the resulting orange solid was centrifuged and washed three times with ethanol.

### 2.4 Synthesis of MIL-100(Fe)-SH

The functionalization of MIL-100(Fe) with thiol groups was conducted following an adaptation of an experimental protocol described in a previous report.^23^ First, 0.4 g of MIL-100(Fe) was activated, inside a three-round-neck flask, at 423 K for 24 h under N_2_ flow to remove the solvent molecules coordinated to the iron centers. Then, 40 mL of anhydrous toluene and 6 mL of a 0.24 M 1,2 ethanedithiol/toluene solution were added, and the mixture was kept under stirring for 12 h at room temperature. The obtained powder was washed with ethanol to remove the unreacted 1,2-ethanedithiol molecules and dried under a vacuum.

### 2.5 Preparation of MIL-100(Fe)-SH/3D devices

The 3D device was designed using the Rhinoceros 6 software (McNeel & Associates, USA) and printed utilizing a Form 2 3D Printer (Formlabs) with Formlabs Clear V4 photoactive resin (FLGPCL04), which is characterized by low cost, smooth surface finish, and fine features and high detail. It consists of methacrylate monomers/oligomers and an initiator. The printing time was between 100 to 254 min to make 1 to 50 devices, respectively, with a 25 μm layer resolution and using 0.25 mL of liquid resin per device. Once printed, the devices were cleaned with 2-propanol to remove unreacted monomers and cured under UV radiation for 4 h. The immobilization of MIL-100(Fe)-SH on the 3D printed device was carried out following an easy coating method based on a previous published report.^37^ Basically, an acetone suspension of MIL-100(Fe)-SH (150 mg of MOF/5 mL acetone) was mixed with 1 g of DMF solution containing 7.5 wt % of poly(vinylidene fluoride) polymer by sonication. A stream of nitrogen gas was applied for acetone removal, and the resulting ink was added drop by drop on the 3D printed support, which was further dried at 333 K for 1 h.

### 2.6 Metal Extraction

The metal adsorption capacity of the developed materials and devices was studied by using 50 mL of a 1 mg L^–1^ heavy metal solution under continuous stirring. After extraction, the metal concentration in the supernatants was determined by ICP-OES (Optima 5300 DV, Perkin-Elmer) and CV/ HG-AFS (AFS-640, Rayleigh). The conditions are summarized in Tables S1 and S2, respectively.

## 3 Results and Discussion

### 3.1 Preparation of MIL-100(Fe)-SH

**Figure 1:**
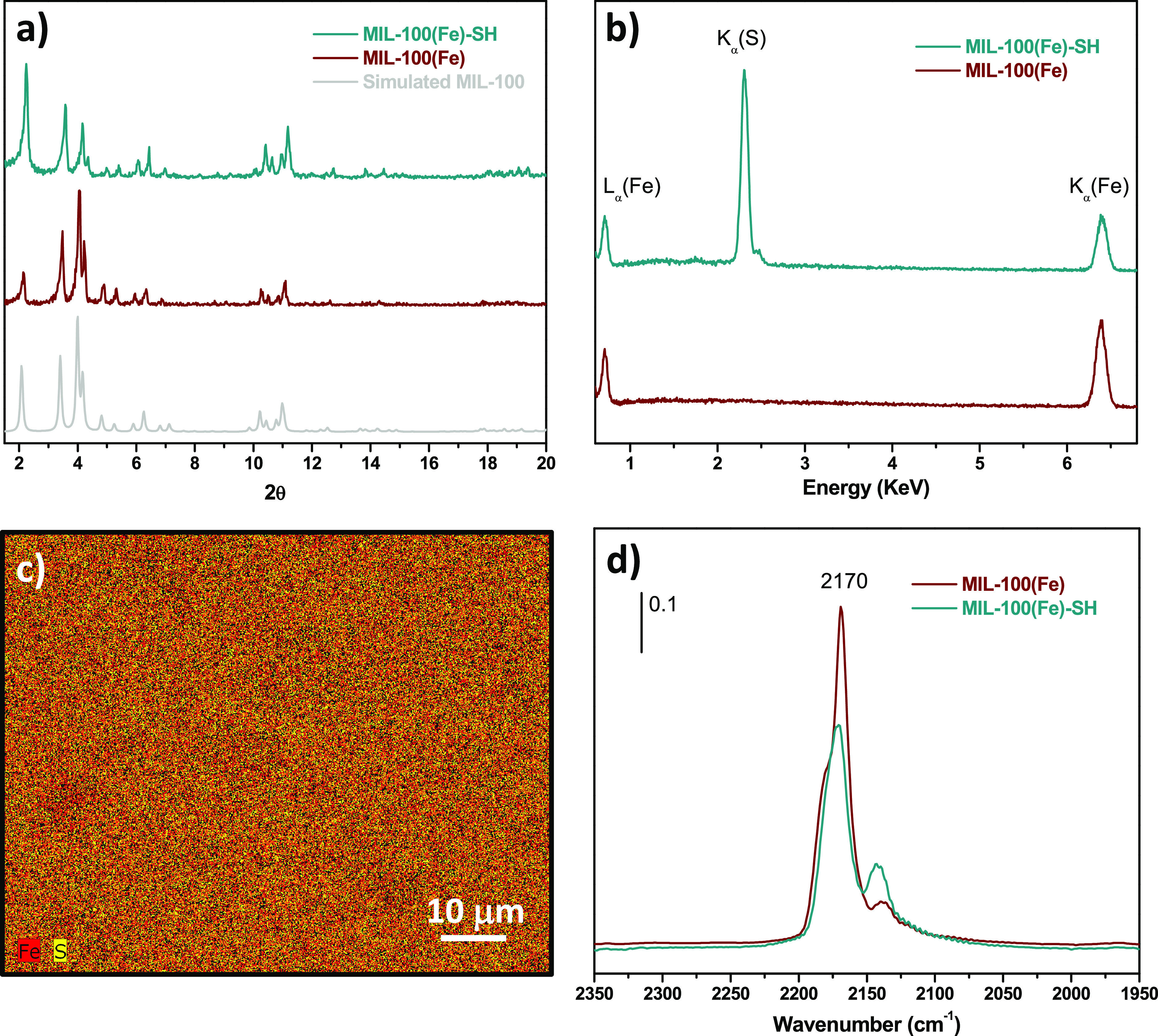
(a) XRD patterns of MIL-100(Fe) and MIL-100(Fe)-SH. (b) EDS spectra of MIL-100(Fe) and MIL-100(Fe)-SH. (c) Fe and S EDS mappings of MIL-100(Fe)-SH. (d) FTIR spectra of CO adsorbed at 100 K on MIL-100(Fe) and MIL-100(Fe)-SH.

The thiol-functionalized MOF was obtained by simple grafting of 1,2-ethanedithiol on the open iron centers of the water-stable and highly porous MIL-100(Fe). The X-ray diffractograms of the as-synthesized MIL-100(Fe) and the thiol-grafted MIL-100(Fe) are shown in Figure 1a. Both materials showed high crystallinity, and the XRD patterns matched well with the simulated one, indicating the preservation of the structure after the grafting process. The incorporation of thiol groups on MIL-100(Fe) was demonstrated by EDS. In comparison with the EDS spectrum of MIL-100(Fe) (Figure 1b), an additional band at 2.3 KeV is observed in the spectrum of the functionalized MIL-100(Fe), which corresponds to the S (Kα) signal. The EDS analysis (Table S3) indicates an S/Fe ratio of 0.80. Furthermore, elemental EDS mapping of MIL-100(Fe)-SH (Figure 1c) shows a homogeneous distribution of S in the material. The functionalization of MIL-100(Fe) to yield MIL-100(Fe)-SH was evidenced by FTIR spectroscopy (Figure S1). The FTIR spectra of both samples show the characteristic IR peaks of MIL-100 MOF at 1626, 1447, and 1380 cm^–1^ that are assigned to the C–O stretching vibration of the carboxylic group and symmetric and asymmetric vibration of the OCO group, respectively.^38^ The two absorption bands around 2970 and 2886 cm^–1^ and the weak band around 2575 cm^–1^ observed in the spectrum of the MIL-100(Fe)-SH sample are attributed to C–H and S–H stretching vibrations of the 1,2 ethanedithiol molecules, respectively, indicating thiol functionalization of the MOF.^39,40^ The grafting of 1,2 ethanedithiol molecules to the open iron centers of MIL-100(Fe) was also studied by FTIR spectroscopy of adsorbed CO at 100 K. The IR spectra of CO adsorbed on activated MIL-100(Fe) before and after functionalization are shown in Figure 1d. Adsorption of CO on MIL-100(Fe) resulted in an asymmetric peak at 2170 cm^–1^, which, in agreement with other authors,^41,42^ is assigned to the stretching vibration of CO adsorbed on the open iron sites. This band is also present in the IR spectrum of CO adsorbed on the functionalized MIL-100(Fe)-SH, although of lower intensity, indicating that the coordinately unsaturated iron centers are partially occupied by the thiol molecules. The morphologies of the prepared samples were investigated by scanning electron microscopy. As can be observed in the corresponding SEM micrographs (Figure S2), both MOFs are formed by aggregates of particles with globular shape, indicating that the functionalization does not alter the morphology of the MOF. TGA analysis was carried out to evaluate the thermal stability of the grafted sample (Figure S3). The TGA curve of MIL-100(Fe)-SH showed a continuous weight loss, attributed to the removal of grafting 1,2-ethanedithiol and solvent molecules, followed by degradation of the framework at about 380 °C, which is similar to that of pristine MIL-100(Fe).

**Figure 2:**
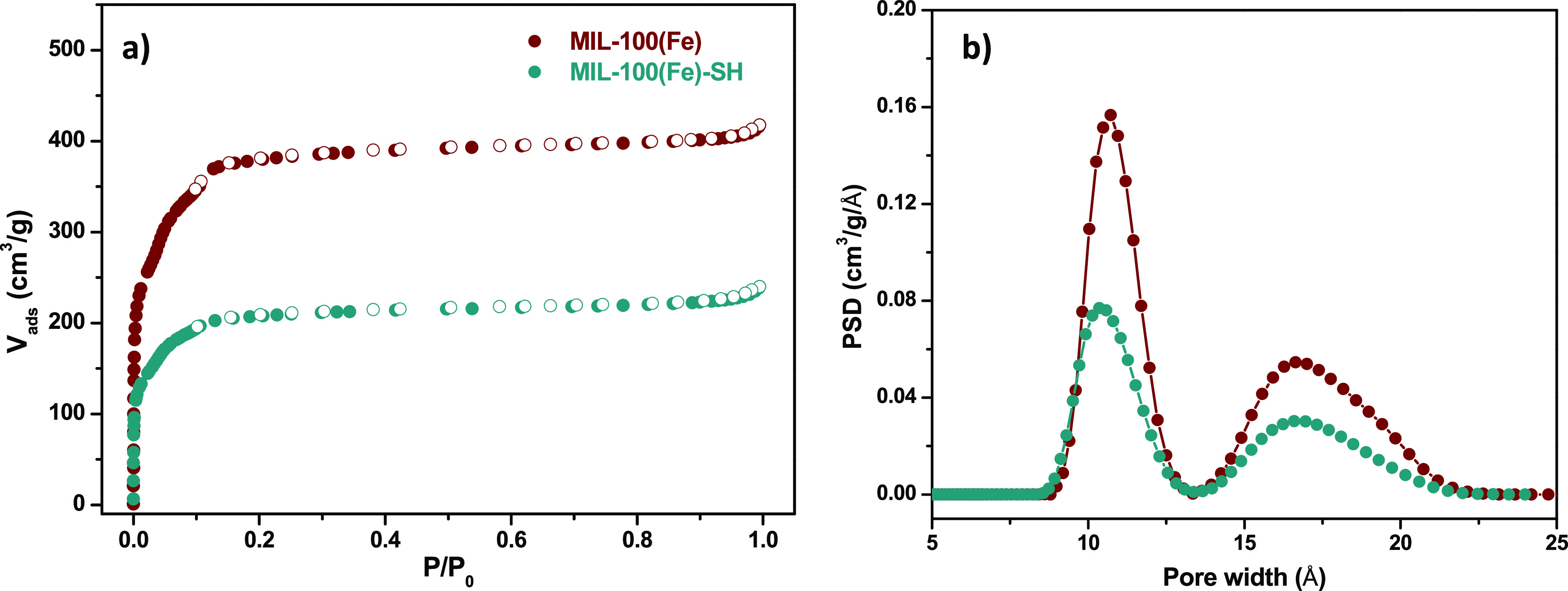
(a) N_2_ adsorption–desorption isotherms and (b) pore-size curves of MIL-100(Fe) and MIL-100(Fe)-SH.

The BET surface area values of MIL-100(Fe) before and after functionalization, obtained from the analysis of the N_2_ isotherms, as well as the corresponding pore volume and pore size values are shown in Table 1. Both materials exhibit a high N_2_ uptake at a relative pressure lower than 0.1 (Figure 2a), indicating their microporous nature, which was confirmed by the pore size distributions (Figure 2b). However, a notable reduction of pore volume and surface area was produced after functionalization, which is due to thiol molecules partially occupying the space inside the pores. Similar results have been previously reported for the functionalization of different MOFs.^43−45^

**Table 1:** Sample Textural Analysis

sample	*S*_BET_ (m^2^ g^–1^)	*V*_p_ (cm^3^ g^–1^)	pore width (Å)
MIL-100(Fe)	1507	0.64	9–12/15–20
MIL-100(Fe)-SH	840	0.36	9–12/15–20

### 3.2 Adsorption of Mercury under Batch Conditions

The Hg extraction capacity of the MIL-100(Fe) and MIL-100(Fe)-SH MOFs was investigated under batch conditions. For that, 1 mg L^–1^ Hg(II) aqueous solution was put in contact with the prepared materials under continuous stirring during 24 h, and the removal capacity was determined using eq 1.
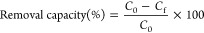
1where *C*_0_ and *C*_f_ are the Hg concentrations before and after extraction. MIL-100(Fe) extracted 78 ± 4% of Hg(II), whereas MIL-100(Fe)-SH reached a 96 ± 5% extraction, demonstrating that, because of the strong soft acid–base interaction between −SH groups (soft base) and Hg (soft acid), the functionalization of the MOF with thiol groups improves the mercury adsorption capacity of the material.^46−49^ The adsorption mechanism was studied by measuring the pH values of the solution before and after the adsorption of Hg(II) by MIL-100(Fe)-SH (Figure S4). The pH of the solution becomes more acidic as the Hg(II) extraction increased, indicating the release of H^+^ ions and the probable ion exchange adsorption process during mercury uptake.^40,50^ In addition, it should be noted that the structure of MIL-100(Fe)-SH after extraction was not affected (Figure S5), indicating the high chemical stability of the functional material.

### 3.3 Characterization of MIL-100(Fe)-SH/3D Printed Devices

**Figure 3:**
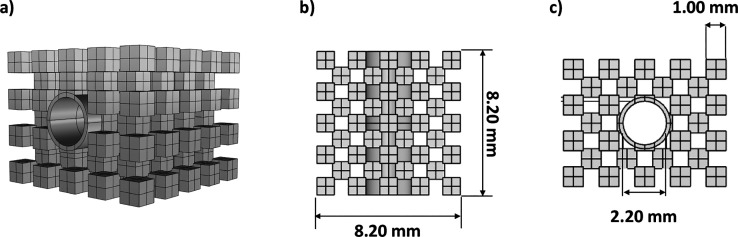
Design of the 3D printed support: (a) perspective view, (b) top view, and (c) front view.

To improve the applicability of the developed adsorbent, it was incorporated on a 3D printed support by a facile coating procedure using an MIL-100(Fe)-SH/PVDF suspension. The 3D support is based on a matrix of interconnected cubes with a cylindrically shaped hole on the center of the device for the incorporation of a magnetic stirrer (Figure 3), which allows its simultaneous use as a stirrer and as sorbent.

**Figure 4:**
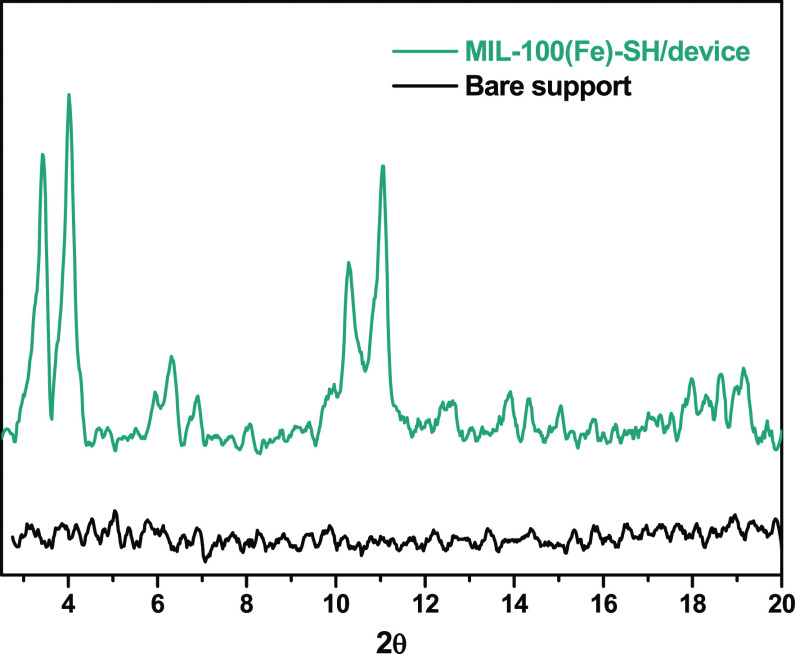
XRD patterns of the 3D support before and after the coating procedure.

**Figure 5:**
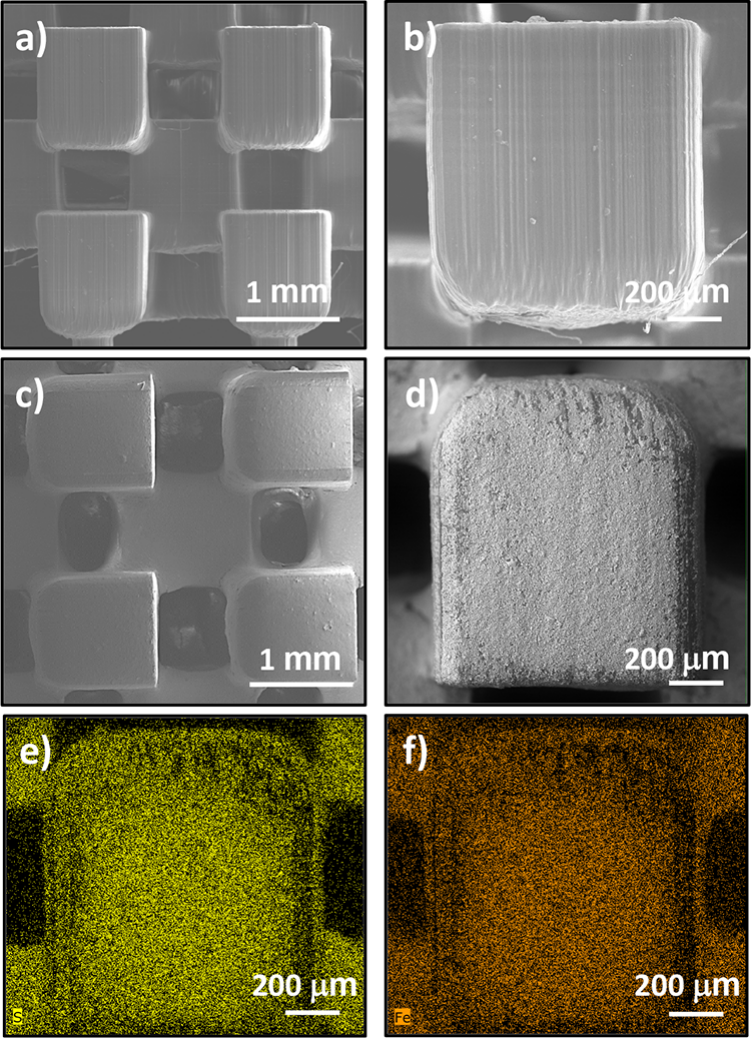
SEM micrographs of the (a, b) bare 3D support and (c, d) MIL-100(Fe)-SH/device at different magnifications. (e) S and (f) Fe EDS mappings of the MIL-100(Fe)-SH/device.

Figure 4 shows the XRD patterns of the bare 3D printed support and after the coating with MIL-100(Fe)-SH. The diffractogram of the bare support does not show any diffraction line; however, the XRD diffractogram of the MIL-100(Fe)-SH/device exhibits the characteristic peaks of the MIL-100(Fe) structure, indicating that the applied coating procedure allows the incorporation of the MOF on the 3D support. The SEM images of the 3D support before and after the incorporation of MIL-100(Fe)-SH are shown in Figure 5. It can be observed that the uncoated support exhibited a smooth surface, which after the coating process appears covered by a uniform layer of MIL-100(Fe)-SH particles. Furthermore, sulfur (Figure 5e) and iron (Figure 5f) mappings showed the homogeneous distribution of both elements on the device, demonstrating the uniform deposition of MIL-100(Fe)-SH on the 3D support. The BET surface area of the MIL-100(Fe)-SH/device determined by N_2_ physisorption was 67 m^2^ g^–1^, which is given by the incorporation of the MOF on the support, as the bare device has zero surface area.

### 3.4 Evaluation of the MIL-100(Fe)-SH/Device in the Extraction of Heavy Metals

**Figure 6:**
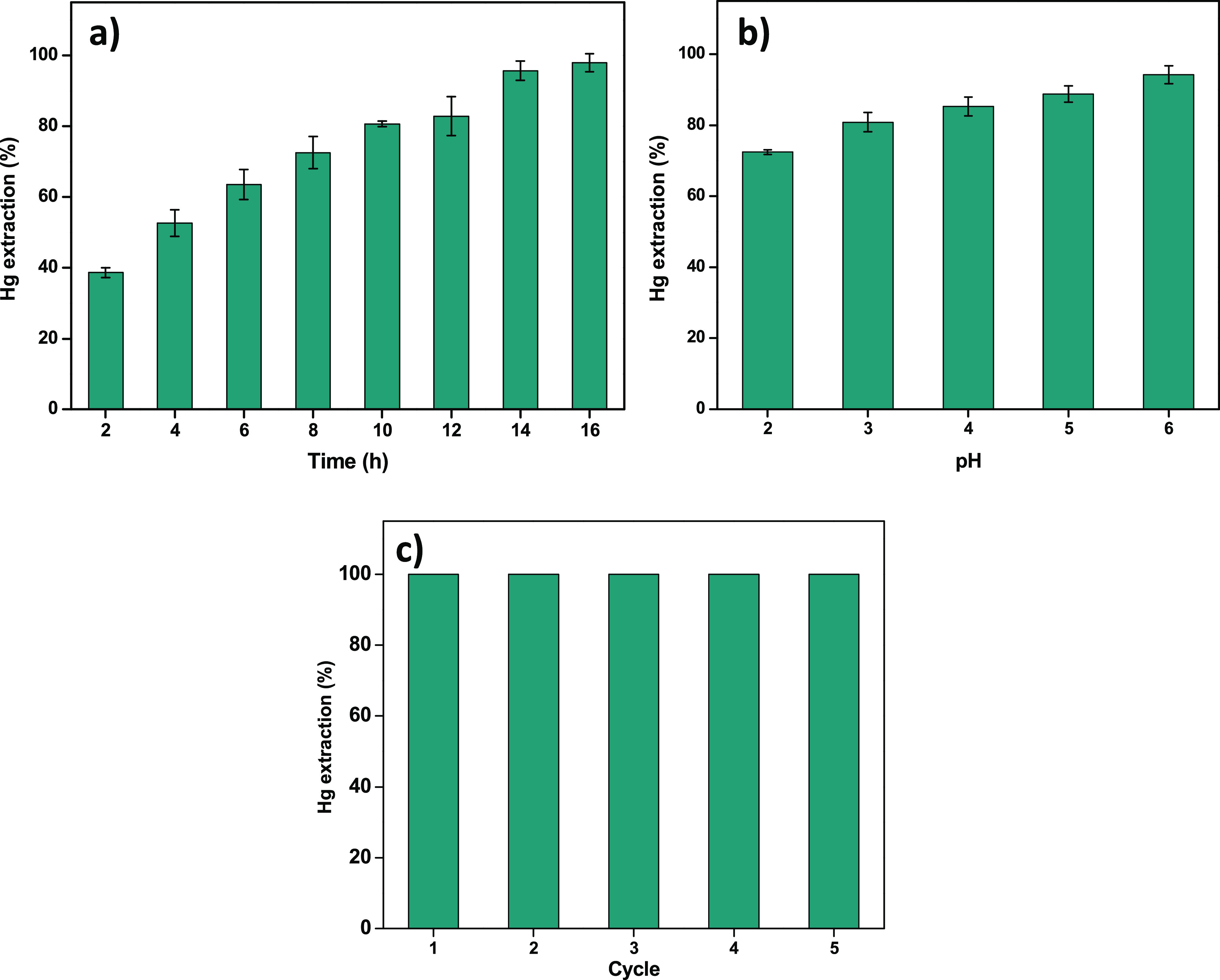
Effect on the Hg extraction capacity of (a) the contact time and (b) the sample pH (1 mg L^–1^, 50 mL, 24 h). (c) Recyclability of the MIL-100(Fe)/device for adsorption of Hg(II) from water.

The developed MIL-100(Fe)-SH/device was tested for the extraction of Hg(II). Figure 6a shows the effect of contact time on Hg(II) removal by the MIL-100(Fe)-SH/device. It can be seen that the removal percentage of Hg(II) increased by increasing the contact time from 2 h (38%) to 16 h (98%), and a further increment of the time did not improve it much. Therefore, 16 h was selected as the optimal extraction time. It should be noted that, at this time, the removal percentage of Hg(II) by a PVDF/device without the MOF was 37%, which indicates that the extraction capacity of the MIL-100(Fe)-SH/device is mainly due to the presence of the functionalized MOF.

In view of the influence of extraction medium pH on the stability of the adsorbent and on its extraction performance, the Fe leaching at the medium and the Hg(II) percentage removal at different pH values were studied. As shown in Figure 6b, the lowest Hg(II) adsorption was obtained at acid pH, which, according to the literature, may be due to the competition between the metal and the H^+^ ions for thiol groups.^51−53^ In addition, under these conditions, a small amount of iron was detected in the supernatant solution (Figure S6), indicating some degradation of the MIL-100(Fe)-SH material. As the pH of solution increased, the extraction percentage of Hg also increased, reaching the Hg(II) maximum adsorption capacity, with a negligible iron release in the solution at pH 6, which is probably due to the fact that, at this pH value, attractive electrostatic interaction can take place between the negatively charged surface of the MOF (Figure S7) and Hg(II). Thus, pH 6 was selected as the optimum pH value. To verify the potential reusability of the MIL-100(Fe)-SH/device, recycling tests were also performed. Between consecutive extractions, the device was washed with 0.1% thiourea–0.01 M HCl solution before reuse. As shown in Figure 6c, the extraction capacity of the MIL-100(Fe)-SH/device was almost identical after five Hg(II) adsorption–desorption cycles, which indicates the excellent reusability of the developed device.

To further evaluate the applicability and versatility of the developed MIL-100(Fe)-SH/device as an adsorbent, it was tested for the simultaneous extraction of Hg(II), Pb(II), and As(V) in tap, well, and lake water samples collected from Chihuahua (Mexico). The results are shown in Table 2. As can be observed, the obtained recoveries for Hg(II) and Pb(II) were 100% for all of them, whereas in the case of As(V), they ranged between 90 and 100% for well and tap water, respectively, which are comparable or even higher than the recoveries of these metals in real water samples reported using other adsorbents,^54−57^ demonstrating the potential of the developed device for the treatment of different water samples polluted with heavy metals. The low recovery of As(V) obtained for the lake water is probably due to the high amount of organic matter present in the sample, which limits the interaction of the metal with the thiol groups of MIL-100(Fe)-SH.^58^

**Table 2:** Analysis of the Recoveries of Hg, Pb, and As in Well, Tap, and Lake Water (*n* = 3)

metal	well water			tap water			lake water		
	*C*_0_	*C*_f_	removal	*C*_0_	*C*_f_	removal	*C*_0_	*C*_f_	removal
	(mg L^–1^)	(mg L^–1^)	(%)	(mg L^–1^)	(mg L^–1^)	(%)	(mg L^–1^)	(mg L^–1^)	(%)
Hg	0.88 ± 0.03	<LD^a^	100	2.37 ± 0.04	<LD^a^	100	0.81 ± 0.08	<LD^a^	100
Pb	19.55 ± 0.93	<LD^a^	100	6.25 ± 0.15	<LD^a^	100	6.11 ± 0.08	<LD^a^	100
As	13.18 ± 1.00	1.18 ± 0.25	91.05	15.07 ± 0.24	<LD^a^	100	64.17 ± 1.23	49.97 ± 2.06	22.13

a Limit of detection.

## 4 Conclusions

In this work, a highly porous thiol-functionalized MIL-100(Fe) MOF was prepared by a simple grafting method. The obtained MOF was used for the dispersive solid-phase extraction of Hg(II) ions, obtaining a high extraction capacity of Hg(II), significantly higher than that of bare MIL-100(Fe), which confirmed the key role of the thiol groups on the adsorption process. Using the prepared thiol-grafted MOF, a functional MIL-100(Fe)-SH/device was obtained by an easy and straightforward coating method with a 3D printed support. The developed device, which simultaneously acts as stirrer and sorbent, could be reused efficiently over five adsorption cycles and showed good performance for the solid-phase extraction of Hg(II), Pb(II), and As(V) in real water samples, achieving recoveries of 100% for two of the ions in the three analyzed samples and demonstrating its excellent features to analyze real water samples.

## Supporting Information Available

Operation parameters of ICP-OES and HG/CV-AFS; EDS analysis of MIL-100(Fe)-SH; FTIR spectra, SEM images, and TGA curves of MIL-100(Fe) and MIL-100(Fe)-SH; pH changes and Fe release after extraction using the MIL-100(Fe)-SH material; and zeta potential and XRD after extraction of MIL-100(Fe)-SH MOF (PDF)
