# Supplementary material for: Thiol-Functionalized MIL-100(Fe)/Device for the Removal of Heavy Metals in Water
Source: Inorg Chem. 2023 Nov 18;62(48):19404–11. doi: 10.1021/acs.inorgchem.3c01544 (PMC10698723; doi:10.1021/acs.inorgchem.3c01544)
Supplement: Supplementary file 1 — ic3c01544_si_001.pdf [file ic3c01544_si_001.pdf]

Supplementary Material

Thiol-functionalized MIL-100(Fe)/device for the removal of heavy metals in water

D.R. Sáenz-García,[a,b] Andreu Figuerola,[b] Gemma Turnes Palomino,[b] Luz O. Leal,[a*] Carlos Palomino Cabello[b*]

[a]Environment and Energy Department, Advanced Materials Research Center (CIMAV) S.C., Miguel de Cervantes 120, Chihuahua, Chih. 31136, Mexico

[b]Department of Chemistry, University of the Balearic Islands, Cra. Valldemossa Km 7.5, 07122, Palma, Spain

*E-mail: luz.leal@cimav.edu.mx

*E-mail: carlos.palomino@uib.es

Table S1. Operation parameters of ICP-OES for Hg, As, Fe, and Pb.

Operation parameters for ICP-OES		
Wavelength (nm)	As	188.979
	Pb	220.353
	Hg	184.886
	Fe	238.204
RF power (W)		1300
Plasma Flow (L min^{-1})		15
Auxiliary gas flow (L min^{-1})		0.2
Nebulizer		concentric
Sample flow (mL min^{-1})		1.5

Table S2. Operating parameters for HG/CV-AFS for Hg, Pb and As.

**Parameter**	**As**	**Pb**	**Hg**
KBH_4 (%)	1.7%	1.7%	0.08%
HCl (%)	10.53%	1.2%	4.5%
Atomizer temperature (°C)	ROOM	ROOM	200°C
Ar flow (mL min^{-1})	300	400	200

Table S3. EDS analysis of MIL-100(Fe)-SH.

**Element**	**Atom-%**
Fe	9.49
S	7.77
C	35.7
O	47.0

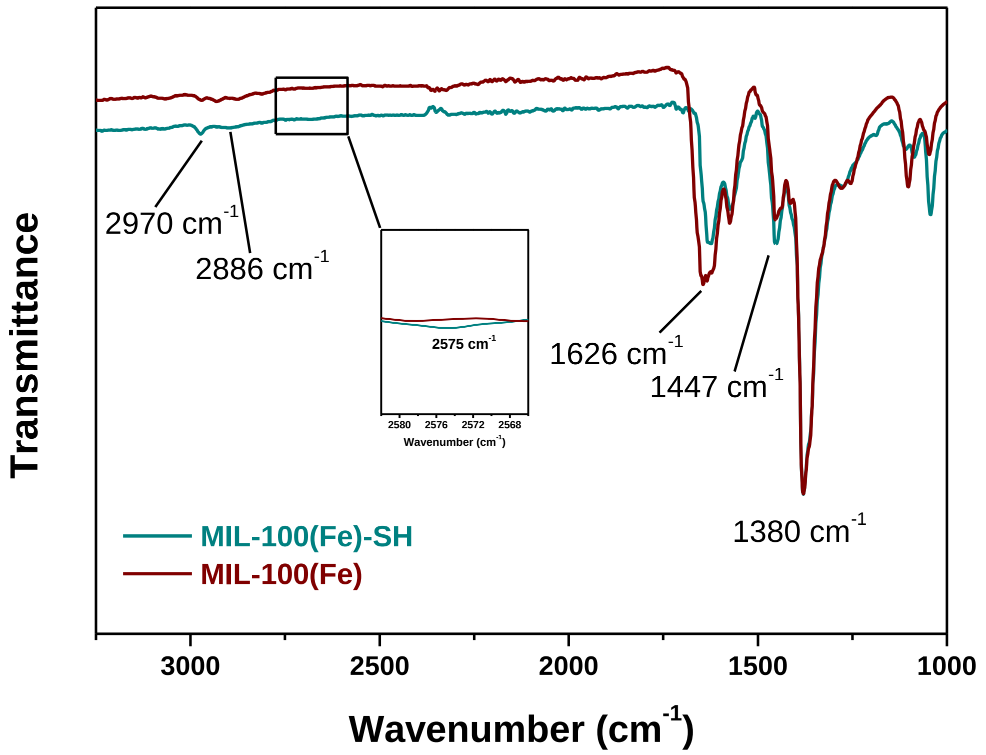

Figure S1. FTIR spectra of MIL-100(Fe) and MIL-100(Fe)-SH.

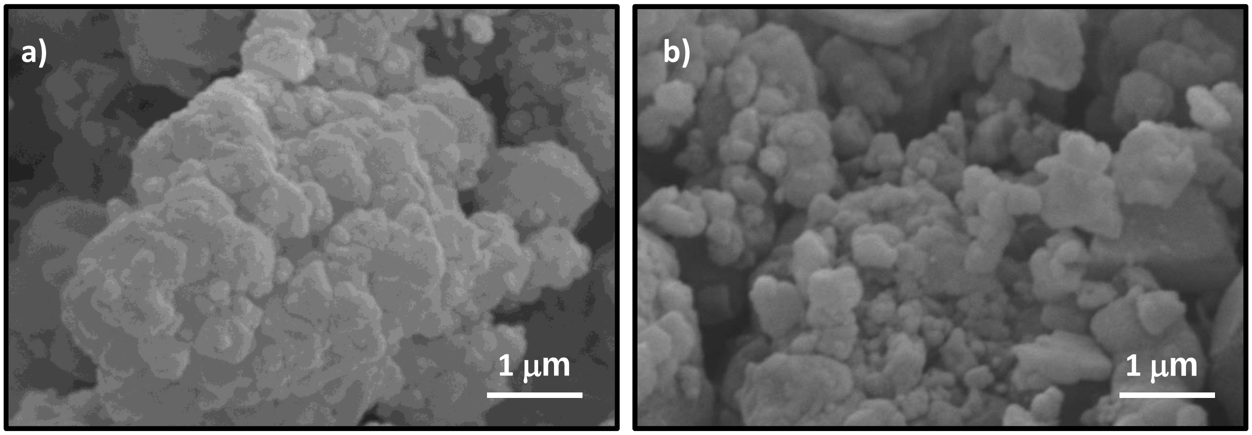

Figure S2. SEM images of (a) MIL-100(Fe) and (b) MIL-100(Fe)-SH.

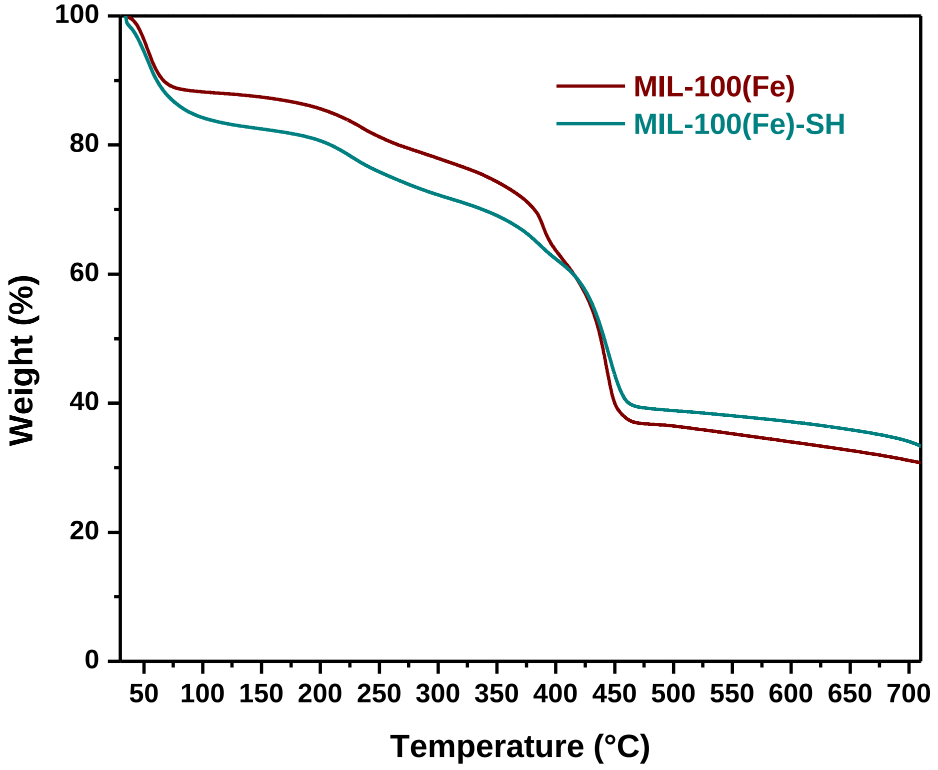

Figure S3. TGA analysis of MIL-100(Fe) and MIL-100(Fe)-SH.

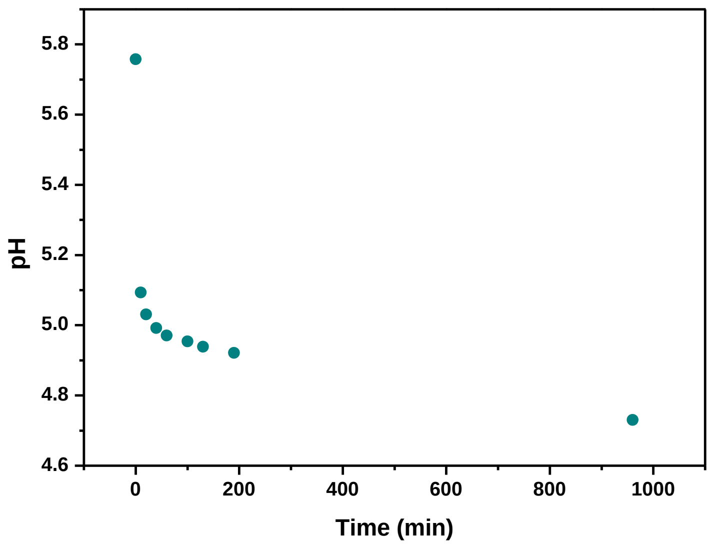

Figure S4. pH of the extracted solution versus time using MIL-100(Fe)-SH as adsorbent. Extraction conditions: 50 mL of mercury solution; C_o = 5 ppm; 125 mg of MOF.

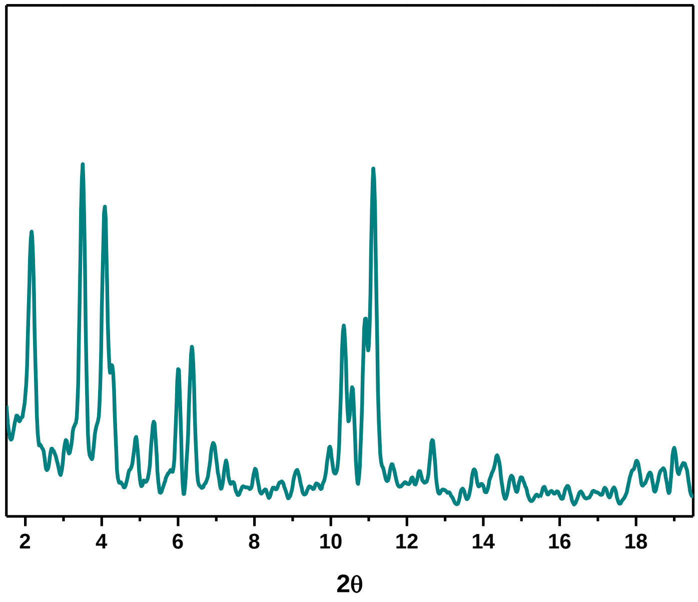

Figure S5. XRD pattern of MIL-100(Fe)-SH after extraction.

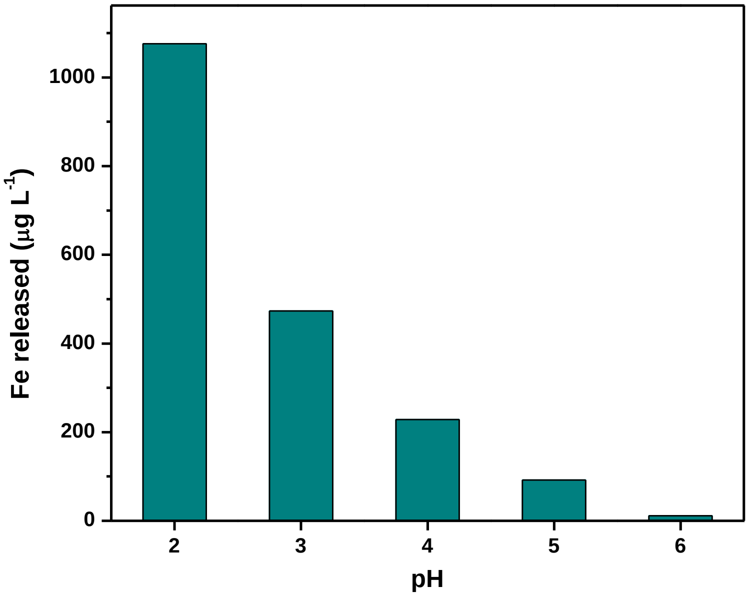

Figure S6. Fe release at aqueous media after Hg(II) extraction at different pH values.

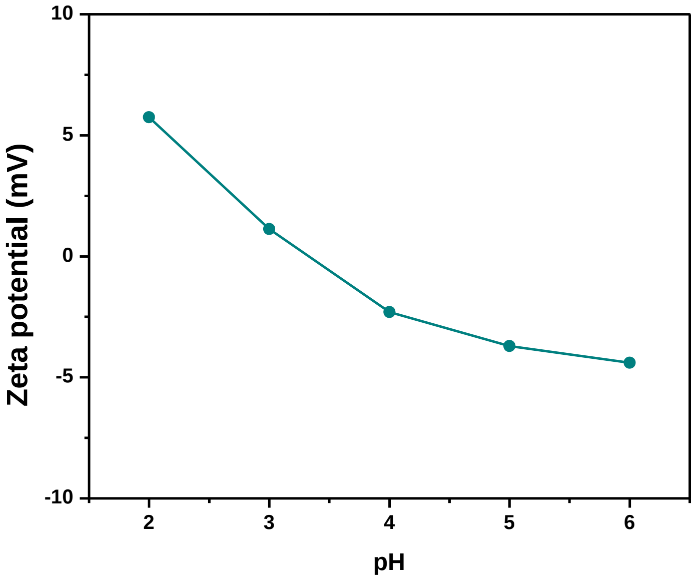

Figure S7. Zeta potential values of MIL-100(Fe)-SH at different pH.
